# The incidence, prevalence and trends of Chronic Kidney Disease and Chronic Kidney Disease of uncertain aetiology (CKDu) in the North Central Province of Sri Lanka: an analysis of 30,566 patients

**DOI:** 10.1186/s12882-019-1501-0

**Published:** 2019-08-28

**Authors:** Asanga Venura Ranasinghe, Gardiye Weligamage Gamini Priyantha Kumara, Ranamuka Henayage Karunarathna, Ambepitiyawaduge Pubudu De Silva, Korale Gedara Dilini Sachintani, Jayaprakara Mudiyanselage Chathurika Nayani Gunawardena, Sembu Kuttige Champika Ruwan Kumari, Mohamed Shali Fathima Sarjana, Janaka Sri Chandraguptha, Mannikawadumesthri Vipula Chandu De Silva

**Affiliations:** 1 Renal Disease Prevention and Research Unit, Ministry of Health, Colombo, Sri Lanka; 2 National Intensive Care Surveillance, Ministry of Health, Colombo, Sri Lanka; 3 Office of Additional Secretary (Development), Ministry of Health, Colombo, Sri Lanka; 4 Department of Pathology, Faculty of Medicine, University of Colombo, Colombo, Sri Lanka

**Keywords:** Chronic kidney disease of uncertain origin (CKDu), CKD/CKDu incidence, CKD/CKDu prevalence, CKDu in Sri Lanka, CKD/CKDu survival rate

## Abstract

**Background:**

Chronic Kidney Disease (CKD) of uncertain origin (CKDu) has affected North Central Province (Anuradhapura and Polonnaruwa districts) of Sri Lanka. The cause is still unknown. The objective of this study was to describe the incidence, prevalence and trend of CKD/CKDu in North Central Province of Sri Lanka.

**Methods:**

A cross sectional survey conducted in North Central Province with GPS mapping in CKDu highly affected areas. The diagnosis of CKD and staging were made according to the Kidney Disease: Improving Global Outcomes paper.

Descriptive statistics used with chi-square test for evaluating dichotomous variables. Log rank test was used to compare survival rates. The population data was obtained from the 2011 Census.

**Results:**

There were 30,566 CKD/CKDu patients in the North Central Province. Incidence of 0.10 in 2009, 0.39 in 2016 in Anuradhapura district, decreased slightly to 0.29 in 2017. Incidence of 0.09 in 2009, 0.46 in 2016 in Polonnaruwa district, decreased slightly to 0.41 in 2017.

The point prevalence in high incidence areas ranged from 2.44–4.35. The 5 year survival rate was 71.2 (Anuradhapura 72.4 and Polonnaruwa 68.3, *p* = 0.0212).

More than 70, 40 and 33% of patients were over 50, 60 and 70 years of age respectively. A male preponderance was seen in all the divisional areas (ranging from 1.3:1 to 2.6:1) and in all the age groups. Farmers were the most affected (70.6% Anuradhapura district and 65.1% Polonnaruwa district). Majority in CKD stage I (4943, 69.6%).

There were 1685 deaths (17.5% of total CKD/CKDu patients, 67.6% of total deaths in CKD/CKDu patients) occurring within the first 3 years of diagnosis.

GPS mapping shows that there is a clustering of households with CKD/CKDu.

**Conclusions:**

The incidence of CKD/CKDu increased up to 2016 with a slight decrease in 2017. The most vulnerable age group was 40 to 60 years. There is a male preponderance. Farmers at a higher risk. Majority were in CKD stage 1. More than two thirds of the deaths of CKD/CKDu patients occurred within three years of diagnosis with disparities in 5 year survival rate among the two districts. There is clustering of cases.

## Background

Chronic Kidney Disease of uncertain aetiology (CKDu) was first reported in the North Central Province of Sri Lanka in the mid 90’s [1]. It is defined as occurrence of Chronic Kidney Disease (CKD) without a known underlying cause [2, 3]. In the affected provinces a significant association has been detected with a rural agricultural population [3, 4]. Most studies reported that males are more affected in numbers and severity [3, 5].

Studies regarding histopathological features in early and late CKDu in Sri Lanka have shown a predominant chronic tubulointerstitial nephritis associated with glomerular scarring, tubular atrophy, interstitial fibrosis and varying degrees of inflammation [5–8]. Some have reported Acute Kidney Injury [9–11]. Several hypotheses were generated and studies have been conducted to find a possible aetiological cause for the disease. These include pesticides [12, 13], fluoride [14], heavy metals such as cadmium and arsenic [4, 12, 15], and hardness of ground water [12, 14], but none have brought forth convincing evidence regarding an aetiological factor.

Data regarding incidence, deaths and changing trends of CKD/CKDu in the highly affected areas is scanty. Early studies showed a point prevalence of 3.7% in Madawachchiya and 3.2% in Huruluwewa divisions [14] but later evidence suggested this to be around 15–23% in Anuradhapura and Polonnaruwa districts [4]. Because of the wide discrepancy of these figures the objective of this study is to describe the incidence, prevalence and trend of CKD/CKDu in North Central Province of Sri Lanka, which was the first province in which the disease was identified.

## Methods

This cross sectional survey of CKD/CKDu patients in North Central Province (Anuradhapura and Polonnaruwa districts) was done in two stages. In the first stage data was retrieved retrospectively using in-ward patient registers and clinic patient registers of the medical wards of the hospitals in the North Central Province for the period from 2003 to 2010 (Anuradhapura district from 2003 to 2010 and Polonnaruwa district from 2006 to 2010). The hospitals included Teaching Hospital Anuradhapura, Base Hospitals of Kabethigollawa, Padaviya, and Thambuththegama and Divisional Hospitals of Madawachchiya and Kahatagasdigiliya in the Anuradhapura district and District General Hospital Polonnaruwa, Base Hospital Madirigiriya and Divisional Hospital Hingurakgoda in the Polonnaruwa district. During the second stage from 2011 to 2016 a list of newly diagnosed patients was prospectively collected from 11 sentinel sites (these included the previously mentioned hospitals and Divisional Hospital Kekirawa from Anuradhapura and Divisional Hospital Bakamuna from Polonnaruwa) on a monthly basis. For all cases from 2003 the diagnosis of CKD/CKDu and the staging of the disease had been made by a Consultant Physician or a Nephrologist. This study does not distinguish between CKD and CKDu. This is because definitive guidelines for distinguishing CKD from CKDu (in Sri Lanka) according to WHO criteria became available only after 2016 [16]. The diagnosis of chronic kidney disease (which includes CKD and CKDu) and staging of the disease in these hospitals had been made according to the Kidney Disease: Improving Global Outcomes paper [17]. The GFR estimations were derived using the CKD-EPI creatinine equation. (2009) [18].

From 2009 onwards the grama niladhari division and the residing village of the CKD/CKDu patients were included in addition to the basic socio-demographic features. The grama niladhari division which is the smallest administrative division in Sri Lanka, comprises three to four villages. Several grama niladhari divisions are grouped together to make a divisional secretariat area (DS division). Several DS divisions together make a district. Anuradhapura district has 22 DS divisions while Polonnaruwa has 7 divisions.

CKD/CKDu patients in the highly affected DS areas in the Anuradhapura (6 DS divisions) and Polonnaruwa districts (4 DS divisions) were prospectively GPS mapped commencing from 2012 onwards using Garmin etrex 10 GPS receiver. In the Anuradhapura district these DS areas included Madawachchiya (2012), Kabethigollawa (2012), Padaviya (2013), Rambewa (2013), Horowpathana (2015) and Kahatagasdigiliya (2015). In the Polonnaruwa district the DS areas included Madirigiriya (2013), Dimbulagala (2013), Hingurakgoda (2014) and Elahara (2014). For this purpose the list of patients diagnosed with CKD/CKDu was obtained from the relevant hospitals. As each DS division comprises several GN divisions, GPS mapping process was focused on GN divisions. Apart from the list, a snowballing method was used to include any CKD/CKDu patients who may have not been identified by the hospital list. The identified CKD/CKDu patients and family members were asked whether they knew of any other residents with similar chronic kidney disease in the neighborhood. The patients detected by this snowballing method were included in the study only if they had medical documentation to confirm CKD/CKDu from any hospital other than those listed previously. Both living as well as dead patients with confirmed medical documentation regarding CKD/CKDu were included for the mapping. During GPS mapping, by questioning the households and subsequently scrutinizing available hospital records (including death certificates in some) an attempt was made to determine if complications of CKD/CKDu was responsible for the death. The exact cause of death was not determined as postmortems had not been done. Staging of CKD among the dead is unknown.

The data collected from hospitals was statistically analysed using SPSS version 10 and STATA 13 student version. Counts and percentages were used to express discrete variables while mean and standard deviation were used to express continuous variables. In univariate analysis, chi-square test was used for evaluating dichotomous variables. For analysis purposes we divided the study population into three groups; from years 2003 to 2008, 2009 to 2012, and 2013 to 2016. This was because the exact date of first diagnosis was not available for the patient population from 2003 to 2008. The group from 2009 to 2012 had an exact date of diagnosis but had been diagnosed at a time when community based CKD/CKDu screening was not organized properly. In 2013 organized community based CKD/CKDu screening commenced in North Central Province. To calculate the incidence, population data was obtained from the 2011 Census [19].

For the calculation of point prevalence and proportion of CKD/CKDu deaths the total number of CKD/CKDu patients obtained during the GPS mapping was used. Point prevalence was defined as the prevalence of living CKD/CKDu patients identified at the time of the GPS mapping in specified DS divisions. Period incidence was defined as the total number of new CKD/CKDu cases occurring during a specific number of years. Log rank test was used to compare survival rates. The population for each DS area was obtained from the 2011 Census [19].

## Results

The study includes a total of 30,566 CKD/CKDu patients who had been diagnosed at eleven hospitals in the North Central Province from 2003 to 2017.

**Fig. 1:**
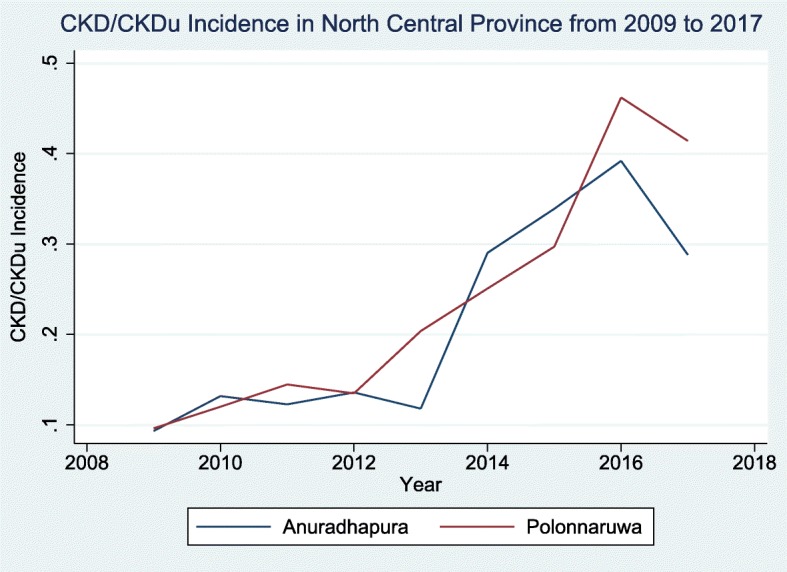
Incidence of CKD/CKDu patients in Anuradhapura and Polonnaruwa districts

The change of incidence from 2009 to 2017 is shown separately for the two districts in Fig. 1. Both districts show a steady increase of CKD/CKDu incidence from 2009 to 2012. The increase was from 0.10 to 0.14 in the Auradhapura district and 0.09 to 0.13 in the Polonnaruwa district. From 2013 to 2016 there was a sharp increase with the incidence in 2016 being 0.39 in the Auradhapura district and 0.46 in the Polonnaruwa district. However in 2017 a decline in the incidence was observed in both districts (Auradhapura 0.29 and Polonnaruwa 0.41).

Table 1 shows the distribution of patients in the three time category periods from 2003 to 2008, 2009 to 2012 and 2013 to 2017. The mean age of the CKD/CKDu patients was 57.2 years with SD ± 13.4 years (Anuradhapura district 57.2 years with SD ± 13.1 years, Polonnaruwa district mean age was 57.4 years with SD ± 14.1 years). In both districts more than 70, 40 and 33% of patients were over 50, 60 and 70 years of age respectively. Only a small percentage of patients (5.4–8.6%) were in the 31 to 40 years age group.

**Table 1:** Socio-demographic characteristics of CKD/CKDu in North Central Province

Characteristics	Anuradhapura (*n* = 20,473)			Polonnaruwa (*n* = 10,093)		
	2003 to 2008 (*n* = 4039)	2009 to 2012 (*n* = 4157)	2013 to 2017 (*n* = 12,277)	2006 to 2008 (*n* = 1474)	2009 to 2012 (*n* = 2011)	2013 to 2017 (*n* = 6608)
	Number (%)	Number (%)	Number (%)	Number (%)	Number (%)	Number (%)
Period Incidence	0.47	0.48	1.43	0.36	0.49	1.63
Sex						
Male	2731 (67.6%)	2796 (67.3%)	7577 (61.7%)	1132 (76.8%)	1477 (73.4%)	4309 (65.2%)
Female	1308 (32.4%)	1361 (32.7%)	4700 (38.3%)	342 (23.2%)	534 (26.6%)	2299 (34.8%)
Age (years)						
Male mean (SD)	56.7 (±13.9)	56.9 (±12.8)	57.7 (±12.2)	56.3 (±14.9)	58.9 (±11.9)	58.0 (±13.6)
Female mean (SD)	56.6 (±15.5)	56.8 (±13.5)	56.8 (±13.3)	53.8 (±17.8)	59.2 (±13.1)	56.0 (±15.3)
Age groups (years)						
< 10	13 (0.3%)	13 (0.3%)	15 (0.1%)	15 (1.0%)	4 (0.2%)	17 (0.3%)
11–20	102 (2.5%)	48 (1.1%)	168 (1.4%)	38 (2.6%)	8 (0.4%)	123 (1.8%)
21–30	100 (2.5%)	95 (2.3%)	205 (1.7%)	45 (3.0%)	27 (1.3%)	176 (2.7%)
31–40	262 (6.5%)	242 (5.8%)	748 (6.1%)	118 (8.0%)	106 (5.3%)	482 (7.3%)
41–50	706 (17.5%)	768 (18.5%)	1989 (16.2%)	261 (17.7%)	307 (15.3%)	1025 (15.5%)
51–60	1229 (30.4%)	1330 (32.0%)	4000 (32.6%)	420 (28.5%)	613 (30.5%)	1890 (28.6%)
61–70	1018 (25.2%)	1118 (26.9%)	3665 (29.8%)	334 (22.7%)	625 (31.1%)	1797 (27.2%)
> 70	609 (15.1%)	543 (13.1%)	1487 (12.1%)	243 (16.5%)	321 (15.9%)	1098 (16.6%)
Occupation						
Farmer	3118 (77.2%)	3174 (76.4%)	5198 (42.3%)	950 (83.3%)	1439 (78.1%)	3116 (47.1%)
Other	921 (22.8%)	983 (23.6%)	2872 (23.4%)	191 (16.7%)	403 (21.9%)	2363 (35.8%)
Not available	0	0	4207 (34.3%)	333 (22.6%)	169 (8.4%)	1129 (17.1%)
Staging of CKD (*n* = 7103)						
Stage I	6	379	3109	242	229	978
Stage II	0	15	108	0	1	44
Stage III	0	289	271	0	6	374
Stage IV	0	165	204	0	0	136
Stage V	0	154	256	0	10	127
Reported Deaths						
Institutions	Not reported	599 (14.4%)^a^	1194 (12.2%)^a,b^	Not reported	Not reported	Not reported

^a^Percentage was calculated by taking the total number of CKD/CKDu for the respective period as the denominator

^b^ 2017 deaths were not available

There was a male preponderance in both districts (male:female ratio 1.8:1 in Anuradhapura; 2.2:1 in Polonnaruwa). This male preponderance was seen in all the DS divisions (ranging from 1.3:1 to 2.6:1) and in all the age groups. It is lowest in the younger age groups (male:female is 1.1:1 in 11–20 years age group in both districts) and highest in 41–60 years age group (male:female is 2:1 in Anuradhapura and 2.4:1 in Polonnaruwa). After 60 years, the male preponderance diminished but still remained high (male:female is 1.8:1 in Anuradhapura and 2.2:1 in Polonnaruwa in > 70 years age category).

Farmers were the most commonly affected occupation group (70.6% from Anuradhapura district and 65.1% from Polonnaruwa district). Details of the staging (Table 1) was available only in a minority of the group (7103, 23.2%). Of this most CKD/CKDu patients were in stage I (4943, 69.6%). Stages II, III, IV and V had 2160 (30.4%) with 547 (7.7%) patients in Stage V respectively.

The incidence of CKD/CKDu from 2012 to 2017 in the DS divisions of both districts is shown in Table 2. Comparison of divisions with period incidence of over 2.40 (six in Anuradhapura district and one in Polonnaruwa district) with those below two shows that there is a statistical difference of period incidence among the two groups of DS divisions of the North Central Province (*p* < 0.001, 95% Confidence Interval for incidence > 2.40 was 2.44–3.63, for incidence < 2.40 was 1.02–1.51).

**Table 2:**
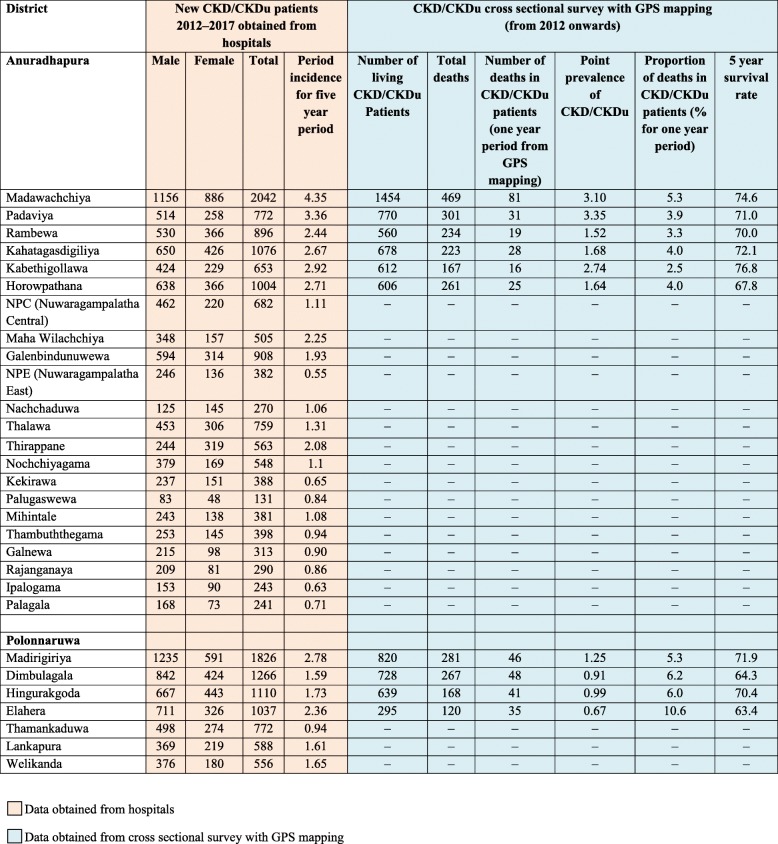
Newly reported CKD/CKDu patients and CKD/CKDu prevalence data

There were 9653 CKD/CKDu patients (6335 from Anuradhapura and 3318 from Polonnaruwa districts) in the 10 DS divisions with the highest incidence which were GPS mapped. The point prevalence of CKD/CKDu in these high incidence areas of Auradhapura ranged from 1.52–3.35 while it was 0.67–1.25 in Polonnaruwa. The 5 year survival rate was 71.2 (Anuradhapura 72.4, 95% CI 71.10–73.7 and Polonnaruwa 68.3, 95% CI 66.1–70.4). Anuradhapura district has a significantly higher survival rate compared to Polonnaruwa district (log-rank test *p* = 0.0212).

Among 9653 patients who were GPS mapped, 2491 (25.8%) were dead patients. This included 1655 deaths, (26.1%) from Anuradhapura and 836 deaths (25.2%) from Polonnaruwa districts. Among these deaths 578 (6% of total CKD/CKDu patients, 23.2% of total deaths in CKD/CKDu patients) occurred within one year of diagnosis (Anuradhapura 396, 23.9%, Polonnaruwa 182, 21.8%) while 1685 (17.5% of total CKD/CKDu patients, 67.6% of total deaths in CKD/CKDu patients) occurred within the first 3 years of diagnosis (1115, 67.4% from Anuradhapura and 570, 68.2% from Polonnaruwa districts). Within the first 5 years of diagnosis 2063 patients were dead (21.4% of total CKD/CKDu patients, 82.8% of total deaths in CKD/CKDu patients). This included 1362 deaths (82.3% of total deaths in CKD/CKDu patients) from Anuradhapura and 701 deaths (83.9% of total deaths in CKD/CKDu patients) from Polonnaruwa districts. It was observed that over 95% of the patients (approximately 2390) did not have identifiable other causes of deaths (such as road traffic accidents, cancer etc.).

**Fig. 2:**
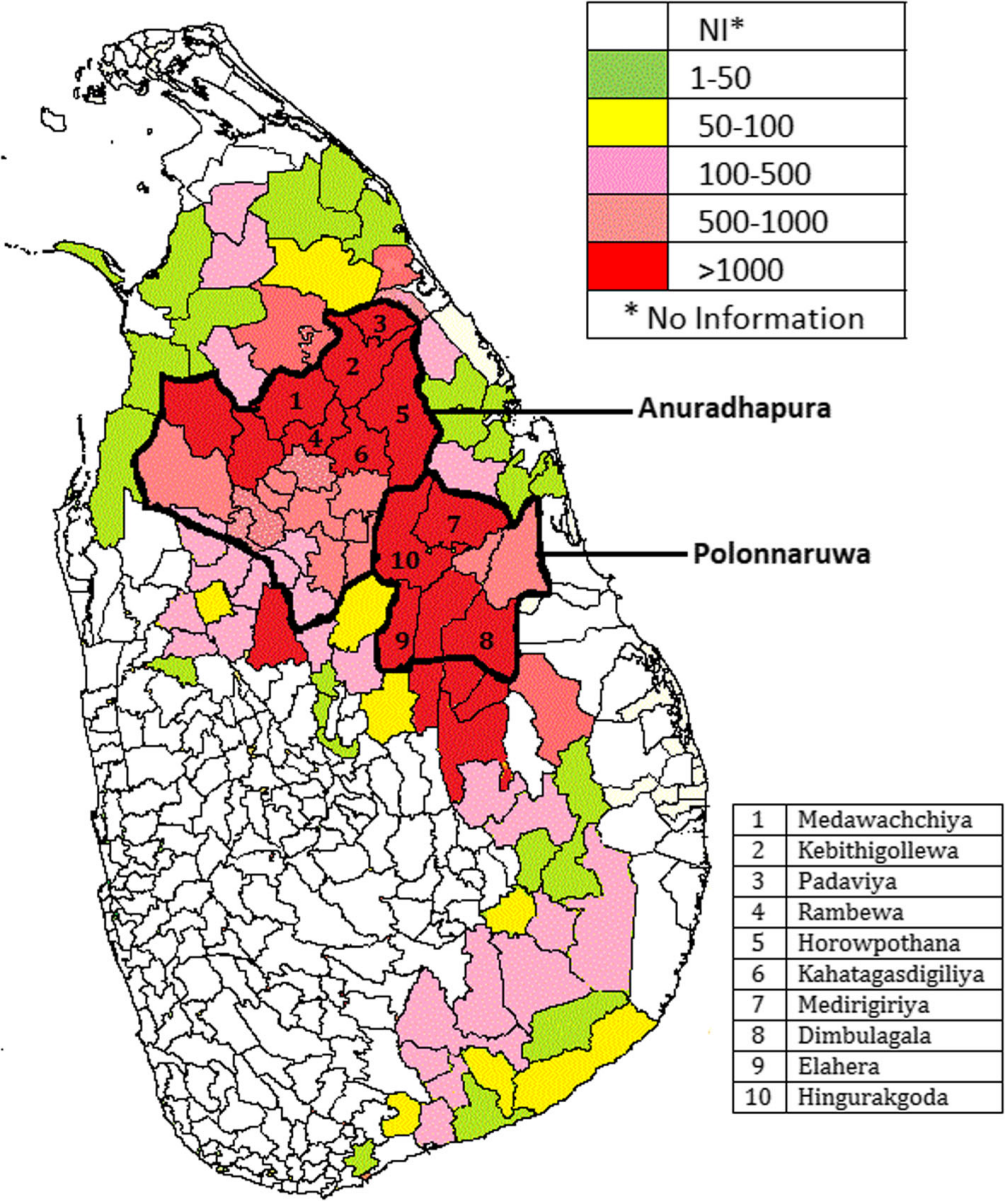
Total number of CKD/CKDu patients in divisional secretariat areas. The map is created based on the data from this study. (This data was collected from hospitals using the same methodology as in this study from other districts)

Figure 2 depicts the total number of CKD/CKDu patients in different DS divisions of Sri Lanka which had been collected using the same methodology as in the present study other than for GPS mapping. This figure shows the seven DS divisions with CKD/CKDu period incidence of > 2.40 in Anuradhapura and Polonnaruwa districts in North Central Province are clustered together. This figure also shows that adjacent DS divisions of neighboring districts also have high numbers.

**Fig. 3:**
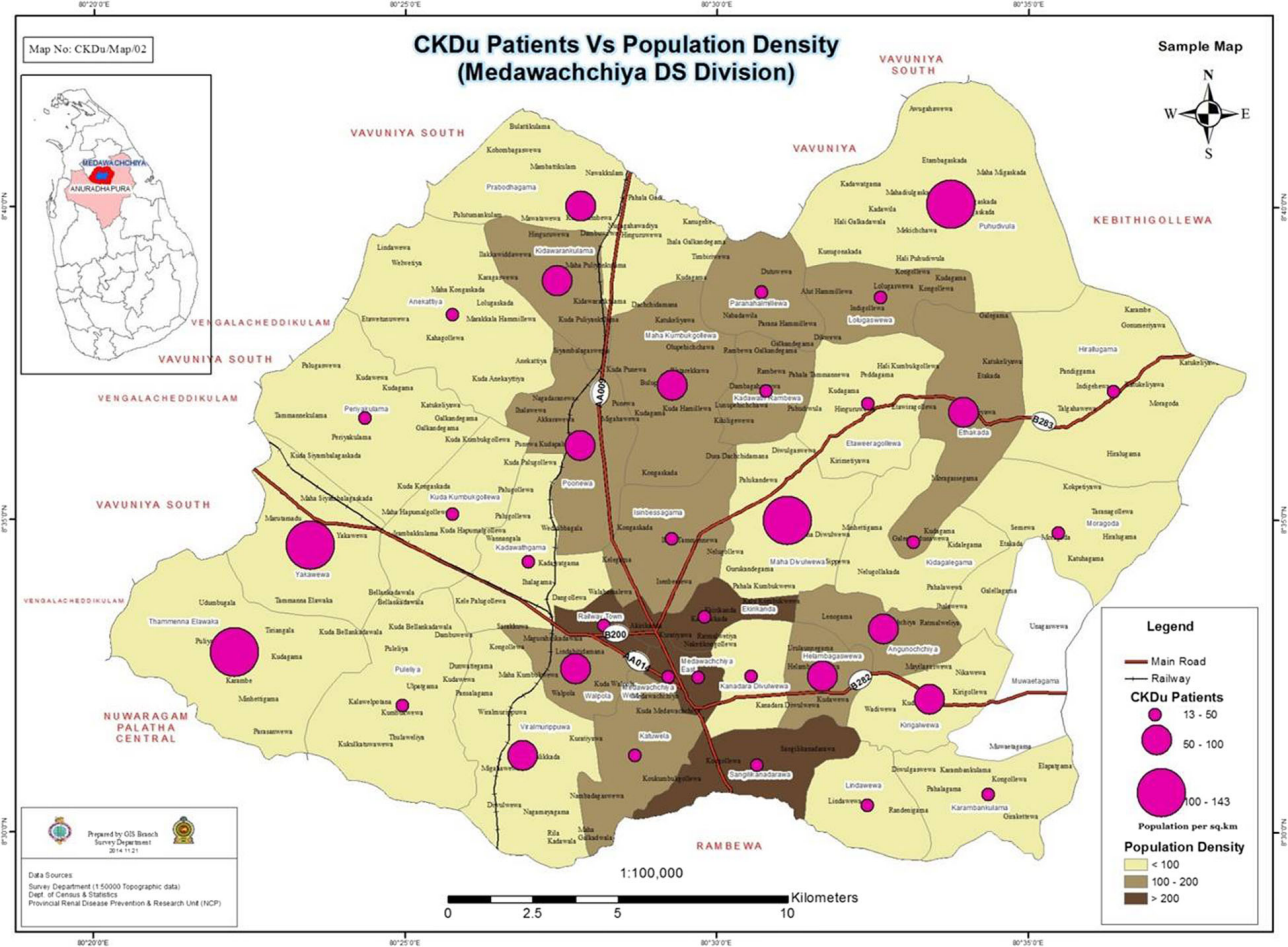
GPS mapping of CKD/CKDu patients vs population density in Madawachchiya DS division of Anuradhapura district. The map is created based on the data from this study. *The high population density GN areas indicated in dark brown are urban settings

**Fig. 4:**
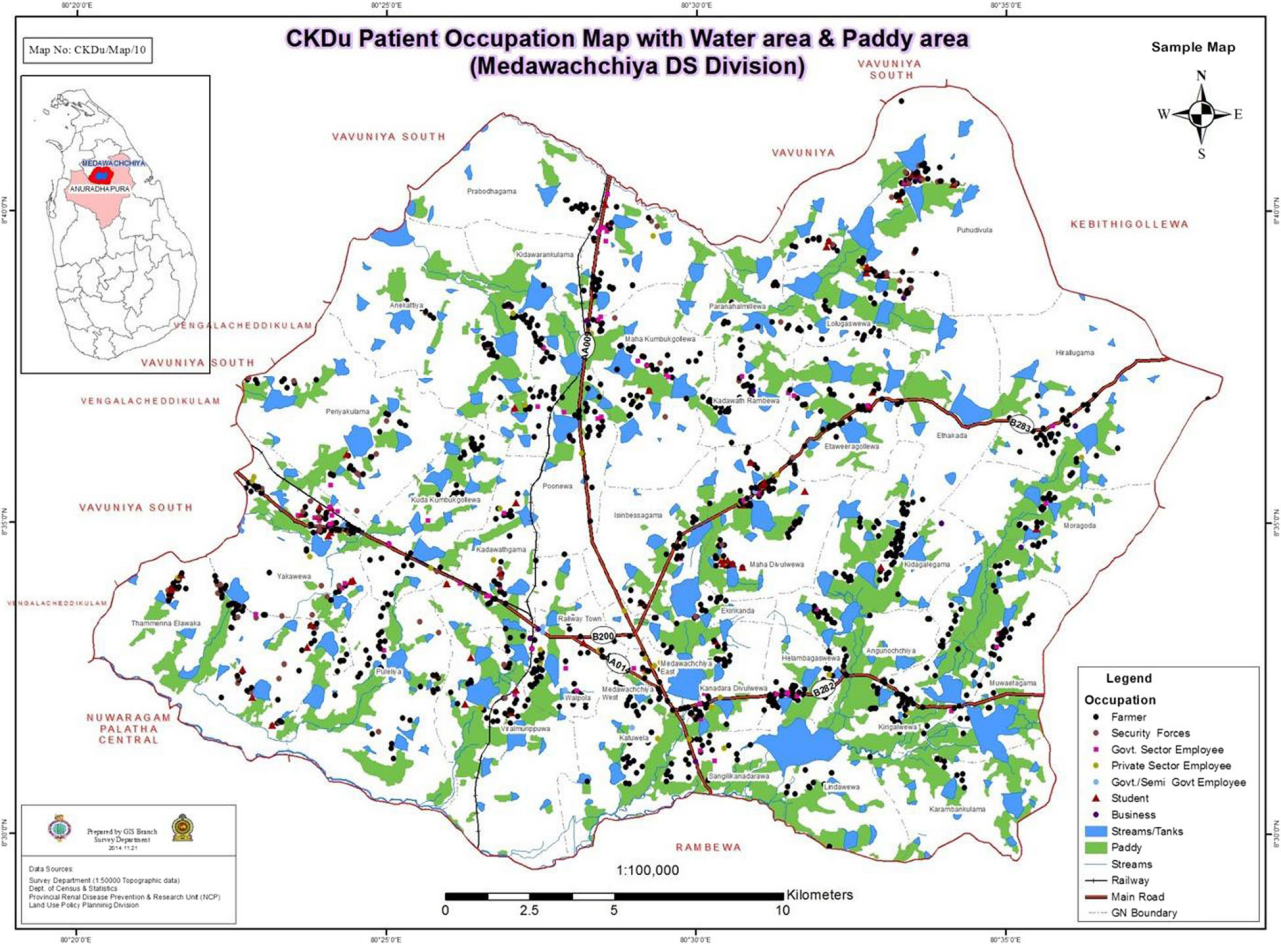
GPS mapping of CKD/CKDu patients’ occupation with water & paddy areas in Madawachchiya DS division of Anuradhapura district. The map is created based on the data from this study

**Fig. 5:**
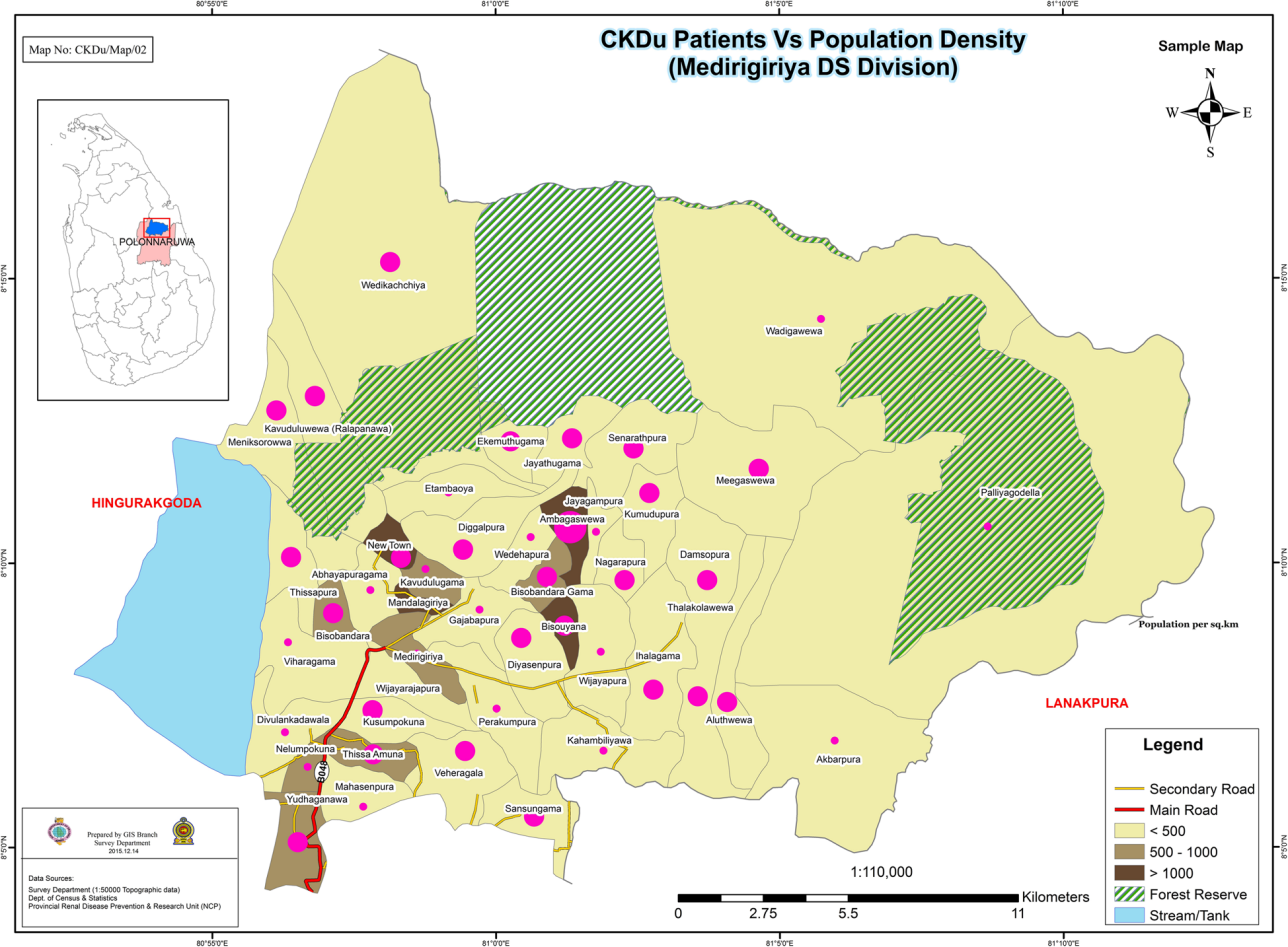
GPS mapping of CKD/CKDu patients vs population density in Madirigiriya DS division of Polonnaruwa district. The map is created based on the data from this study

Figures 3, 4 and 5 shows GPS mapping of CKD/CKDu patients in the two DS divisions with the highest prevalence in Anuradhapura and Polonnaruwa districts. This shows that there is a clustering of households with CKD/CKDu within DS divisions with the highest prevalence.

## Discussion

Our study shows that the incidence of CKD/CKDu in North Central Province increased upto 2016. One reason for the rapid increase in the incidence of the disease in both districts in North Central Province after 2012 may be the organized community based screening programmes conducted by the health sector resulting in increased case detection. Other reasons include increased exposure to aetiological factor or factors which have not been convincingly identified as of yet. This study also shows that the incidence has declined slightly in 2017 in both districts. A probable reason for this may be the steadily increasing provision of safe drinking water in the affected areas [20].

The Balkan Endemic Nephropathy (BEN) thought to be caused by environmental contaminants has shown fluctuating incidence rates since its discovery in 1956 [21]. However its prevalence has remained 3–7% over the last five decades [22].

In the present study the point prevalence from GPS mapping ranged from 1.52 to 3.35 in Anuradhapura and 0.67 to 1.25 in Polonnaruwa. These results are in agreement with the study done by Chandrajith et al. (2011) which showed a point prevalence of 3.7% in Madawachchiya and 3.2% in Huruluwewa [14] but not with the study done by Jayathilaka et al. (2013) which showed a point prevalence of 15–23% [4]. A possible explanation for this is the flows in the design, interpretation and conclusion of the study as shown by Jayasumna et al. (2013) [13].

Our data shows that in the three time categories (2003–2008,2009-2012,2013–2017) the age groups that were most affected were older patients (50 to 60 years and > 60 years). There was no increase within different age groups during the time periods that were studied. As this study does not distinguish between CKD and CKDu it could be argued that the increased incidence in elderly may be due to diabetic and hypertensive nephropathy. However previous studies have demonstrated that diabetes and hypertension contribute to only a minority of CKD in this region [3].

If the majority of patients in this study had CKDu, possible explanations to the increased incidence in older patients include an aetiological factor with a cumulative effect or an aetiological factor which had a prolonged period of pathogenesis. However if this was true, in the later time periods between 2009 to 2012 and 2013 to 2017 there should have been some increase in the proportion of CKD./CKDu in other age groups as well especially if a common environmental factor was responsible. The other possible explanation is that the elderly people are more susceptible to an aetiological factor or factors due to the physiological reasons or co-morbidities.

BEN shows a low incidence in younger age groups and higher incidence after 65 years with the onset between 40 to 60 years [22]. It has a long latent period and thus affects the exposed at a later part of their life [21]. Mesoamerican Nephropathy also shows a high proportion of affected individuals between 50 to 70 age group [23].

Our study shows an increase proportion of male patients within the different age groups and in the different DS divisions. Explanation for this male preponderance includes predominant exposure to the risk factors such as pesticides in farmers or presence of disease modifying factors (eg. use of alcohol and smoking). The possibility of a protective role by female hormones is unlikely as the male:female is lower in the female reproductive age groups. In both districts majority of patients were farmers. However majority of the people residing in these areas are anyway farmers [19]. Explanation for farmers being more affected may be due to higher exposure to aetiological agents such as pesticides. Figure 4 confirms that patients are clustered around areas with paddy fields. This is further confirmed in Figs. 3 and 5 which shows that GN divisions with a high population density which are of urban settings do not have high number of CKD/CKDu patients. Thus clustering pattern within DS divisions is not related to population density.

In BEN both males and females are equally effected [21]. However similar to CKDu in Sri Lanka it was seen among the rural farming population [22]. The Mesoamerican Nephropathy also has similarities to CKDu in Sri Lanka with the disease being commoner among males and older age groups and agricultural workers [23].

Our study also shows that the incidence is not uniform among all DS divisions of the two affected districts in North Central Province. There were six DS divisions in Anuradhapura and one from Polonnaruwa that reported a period incidence of over 2.4. These high incidence areas are clustered closer to each other. This is also apparent from Fig. 2 which shows the geographical distribution of DS divisions that had reported more than 1000 CKD/CKDu cases from 2003 to 2017. These high incidence DS divisions in North Central Province border DS divisions with high number of CKD/CKDu patients in the adjoining districts. Figure 2 indicates that the number of reported CKD/CKDu patients decrease steadily beyond the high incident areas. Thus the effect of the causative factor may be distributed in a gradient fashion with high incidence areas having the most exposure to the causative factor while the lower areas probably have lower exposure. The other explanation may be that the high incidence areas may have other factors that contribute to the causation of disease after initial exposure to a toxic agent.

GPS mapping indicates clustering of cases in both high and low incident areas. According to the maps there are high prevalent areas and low prevalent areas even within a GN division. When the maps are superimposed with paddy fields and irrigation tanks as shown in Fig. 4, this clustering seems to be mainly among the farming communities and around irrigation tanks. The irrigation tanks form a cascading system with water flowing from higher levels to lower levels. While conducting the study we noticed clustering of CKD/CKDu patients towards the lower part of the tanks. Previous studies have also identified clustering of cases towards the lower altitude of the tanks [24].

Similar findings are seen in BEN which exhibit focal occurrence with affected and spared households in close proximity within the same village [25]. BEN was reported to have numerous members of one or several generations in a single household [26].

To the best of our knowledge this is the first publication indicating death rates and 5 year survival rates of CKD/CKDu patients in CKDu affected areas in Sri Lanka. Results from the GPS mapping shows that a total of 2491 (25.8%) out of 9653 CKD/CKDu patients died. Although the exact cause of death was not explored in detail, during the GPS mapping, on discussion with the households and scrutinizing available hospital records (including death certificates in some), it was observed that over 95% of the patients (approximately 2390) did not have identifiable other causes of deaths (such as road traffic accidents, cancer etc.) and therefore were presumed to have died of complications resulting from CKD/CKDu. In comparison hospital based data shows 1793 deaths among 16,434 patients with a death rate of 10% (refer Table 1). This highlights the fact that the implications of CKDu in relation to mortality may be more serious than what is indicated in hospital based statistics.

This study showed a significantly high (log-rank test *p* = 0.0212) 5 year survival rate in Anuradhapura (71.2%) compared to that of Polonnaruwa (68.3%). One possible explanation is the lack of access to health care facilities in Polonnaruwa compared to Anuradhapura based on the assumption that majority of patients died due to CKD/CKDu related causes.

Results from the GPS mapping shows that 6% of total CKD/CKDu patients died within the first year of diagnosis whilst 17.5% died within three years. This represents 67.7% of total deaths in CKD/CKDu. This emphasizes the immediate need for hospital with facilities for dialysis and ideally renal transplants.

This study is limited by the fact that a distinction was not made between CKD and CKDu as data on co-morbidities was not collected. However a previous study has demonstrated that diabetes (2%) and hypertension (14%) contribute to only a minority of CKD/CKDu in this region [3]. Another study has shown that the prevalence of diabetes in North Central Province during the same period was 9.6% [27]. Based on these previous documentations it seems unlikely that diabetes and hypertension would have contributed to major proportion of CKD in the region.

## Conclusion

The incidence of CKD/CKDu in North Central Province has increased up to 2016 with a slight decrease in 2017. A possible explanation for this may be the provision of safe drinking water. The most vulnerable age groups are from 40 to 60 years. There is a male preponderance. Farmers seem to be at a higher risk. Limited data shows that majority of patients are in CKD stage 1.

The 5 year survival rate was 71.2. Anuradhapura district (5 year survival rate 72.4) has a significantly higher survival rate compared to Polonnaruwa district (5 year survival rate 68.3). Among the deaths in CKD/CKDu patients 578 (6% of total CKD/CKDu patients, 23.2% of total deaths in CKD/CKDu patients) occurred within one year of diagnosis while 1685 (17.5% of total CKD/CKDu patients, 67.6% of total deaths in CKD/CKDu patients) occurred within the first 3 years of diagnosis. Within the first 5 years of diagnosis 2063 patients were dead (21.4% of total CKD/CKDu patients, 82.8% of total deaths in CKD/CKDu patients).

Areas reporting higher incidence of CKD/CKDu are clustered together. GPS mapping shows that even within the high incidence areas there is geographic clustering. The clustering was mainly around paddy fields and irrigation tanks.

## Data Availability

Some of the aggregated data are available at the renal registry https://nicst.com/iframe-renal-dev/ while all aggregated data used for this study are available from the Renal Disease Prevention and Research Unit, Ministry of Health, Sri Lanka. The data can be obtained on reasonable request with the permission from the Director General of Health Services, Ministry of Health, Sri Lanka.

## Abbreviations

BEN: Balkan Endemic Nephropathy
CKD: Chronic Kidney Disease
CKDu: Chronic Kidney Disease of uncertain aetiology
DS: Divisional Secretariat
